# Nurse Practitioner Care for Hospitalized Children: A Scoping Review Protocol

**DOI:** 10.3390/nursrep16060179

**Published:** 2026-05-25

**Authors:** Juliana Choueiry, Catherine Chong, Stephanie Lemay, James S. Hutchison, Megan Greenough

**Affiliations:** 1 Children’s Hospital of Eastern Ontario, Ottawa, ON K1H 8L1, Canada; jchoueiry@cheo.on.ca (J.C.); cchong@cheo.on.ca (C.C.); slemay@cheo.on.ca (S.L.); jhutchison@cheo.on.ca (J.S.H.); 2 Children’s Hospital of Eastern Ontario Research Institute, Ottawa, ON K1H 5B2, Canada; 3 Faculty of Health Sciences, School of Nursing, University of Ottawa, Ottawa, ON K1H 8M5, Canada

**Keywords:** nurse practitioner, advanced practice nursing, pediatrics, hospitalized children, acute care, inpatient, scoping review, role operationalization, role implementation

## Abstract

**Background**: Nurse Practitioners (NPs) are increasingly integrated into pediatric inpatient teams in response to evolving healthcare needs and workforce challenges. However, evidence describing how NP roles are operationalized, implemented, and sustained in general pediatric ward settings remain fragmented. **Objective**: This scoping review will synthesize international literature on NP roles in general pediatric inpatient settings, with a focus on determinants of role implementation. Guided by the Participatory, Evidence-based, Patient-focused Process for Advanced Practice Nursing (PEPPA) framework, this review will examine implementation strategies, outcomes, timelines, facilitators, barriers, and required resources to inform future integration and evaluation. **Methods**: This scoping review will be conducted in accordance with Joanna Briggs Institute (JBI) methodology and reported following PRISMA-ScR guidelines. A PRESS-reviewed search strategy will be applied to Scopus, MEDLINE, PsycInfo, CINAHL, Cochrane Central, ProQuest, grey literature, and reference lists for English and French language studies published from 2016 onward. Studies relating to NP roles in general pediatric wards internationally will be included. Data extraction and synthesis will be structured using the PEPPA framework’s implementation domain. Findings will be summarized descriptively and presented in tables and narrative synthesis. **Conclusions**: A targeted synthesis focused on determinants of NP role implementation in this context is needed. This review will clarify how NPs roles have been integrated in general pediatric wards, highlight enabling and constraining factors, and will identify gaps to guide future research, practice redesign, and policy development.

## 1. Introduction

Nurse Practitioners (NPs) are registered nurses educated at a master’s or doctoral level with advanced clinical competencies that allow for an expanded scope of practice [1]. Their role includes diagnosing, treating, and managing various health conditions, ordering and interpreting diagnostic tests, referring to specialists, and engaging in leadership, research and educational activities [1]. Over time, NPs have become integral members of healthcare teams in primary and acute care, supporting innovative models of care that respond to health system needs [2,3]. Over the past two decades, the role of advanced practice nurses (APN), including NPs, has expanded substantially worldwide, with several countries having more established APN systems, including Canada, the United States, the United Kingdom, Australia and Ireland [4]. According to the International Council of Nurses (ICN), the global need for APN roles stems from rising chronic disease burden, a shortage of physicians and healthcare workers, increasing healthcare complexity and a demand for more accessible and affordable healthcare [4].

For instance, in Canada, the NP role originated in the 1960s during a period of chronic physician shortages to improve access to primary care in underserved and remote areas [2,3]. By the 1980s and 1990s, NPs had expanded into hospital settings, particularly neonatal intensive care units (NICUs), where they supported continuity of care for critically ill infants and addressed resident coverage gaps [3]. Similarly, in the United States, neonatal nurse practitioners (NNPs) became key members of NICU teams in response to increasing patient complexity, provider shortages and lack of continuity in care in the 1970s [5]. NNPs utilized their nursing background to assess, manage, and support hospitalized neonates and their families through a holistic lens [3]. These early hospital-based NP roles demonstrated the unique value of advanced practice nursing within interprofessional teams and set the stage for NP role expansion into other pediatric specialties and inpatient settings [3,5].

Children admitted to hospitals today often have complex medical needs that necessitate coordinated, family-centred, and multidisciplinary care [6]. NPs are well-positioned to meet these needs by providing continuity of care, coordinating multidisciplinary management, supporting families, and enhancing patient safety and quality outcomes [2]. The integration of NPs into pediatric acute care teams has the potential to improve patient and family experiences, optimize resource utilization, and mitigate workforce shortages [7]. However, the integration of NP roles remains hindered by persistent systemic and organizational barriers that threaten retention and impact patient outcomes and healthcare sustainability [8,9].

Understanding how NP roles are implemented in pediatric hospitals is essential for workforce planning and the development of sustainable practice models. This is crucial because NP role integration has historically been inconsistent and challenging [9]. Lack of standardized education requirements, unclear role expectations, variable provincial legislation, and limited organizational support have hindered widespread and sustainable implementation [9]. In some cases, NPs have reported resigning within 2 years of practice due to insufficient interdisciplinary collaboration and role clarity [2].

To better understand this topic, an initial exploratory preliminary search in CINAHL, PsycINFO, MEDLINE, the Cochrane Database of Systematic Reviews, Open Science Framework and JBI Evidence Synthesis was conducted using broad terms related to inpatient setting, nurse practitioners and pediatrics. The search retrieved a total of 1916 documents, as illustrated in Table 1. Based on the titles and abstracts of articles in this initial search, very few studies were related to nurse practitioners’ roles in general pediatric wards. The existing studies vary in design, scope, and practice setting. Most of the literature is fragmented and focuses on neonatal practice, subspecialty care, outpatient care, or single-institution experiences, limiting understanding of NP contributions and role implementation across general pediatric wards. No current or ongoing systematic or scoping reviews on the topic were identified in this search.

There are clear gaps in the literature on the NP role in the general pediatric ward setting. Based on the International Council of Nurses’ report on APNs, there is also a global need for clearer standards, regulation, education, and policy to support APN role implementation [4]. The ICN report provides a broad overview and does not specifically address the NP role in the general pediatric inpatient setting. A synthesis of the existing literature is therefore warranted to understand how NP roles are implemented in general pediatric ward settings, including the models of care employed, populations served, and barriers and facilitators influencing implementation.

Thus, this scoping review aims to synthesize the existing evidence on NP practice in general pediatric ward settings internationally. The study will be framed by the implementation domain of the Participatory, Evidence-Based, Patient-Centered Process for Advanced Practice Nursing (PEPPA) Framework, which will guide the organization and synthesis of evidence on how NP roles are planned, integrated, and sustained in pediatric inpatient settings [10]. The overarching goal is to produce an implementation-focused evidence map that identifies determinants, barriers, facilitators, and strategies influencing NP role integration, and to highlight priority areas for future research, policy and practice. This knowledge can be used to contribute to the development of an implementation model specific to this context.

This scoping review will address the following primary question: “What is known about the implementation and practice of nurse practitioner roles in general pediatric ward settings internationally?”. This scoping review also aims to address the following sub-questions:

- What models of NP care have been implemented in general pediatric ward settings, and which patient populations and clinical areas do they serve?
- What is the scope of NP practice in providing care to hospitalized pediatric patients?
- What barriers and facilitators influence NP role implementation and sustainability?
- What outcomes (patient, provider, and system-level) are associated with NP care?

## 2. Materials and Methods

This scoping review will be conducted in accordance with Joanna Briggs Institute (JBI) methodology and reported following PRISMA-ScR guidelines [11,12].

### 2.1. Inclusion and Exclusion Criteria

Sources will be included if they examine NP roles in general pediatric ward settings. Eligible sources must report on one or more of the following aspects: (1) NP model of care on general pediatric wards, (2) NP scope of practice on general pediatric wards, (3) NP role on general pediatric wards and/or (4) patient populations served by NPs on general pediatric wards.

Only literature explicitly describing roles held by NPs, including internationally recognized NP-equivalent advanced practice nursing titles with prescriptive authority and autonomous or semi-autonomous clinical practice, will be included. Sources focusing on Clinical Nurse Specialist (CNS) roles will be excluded, including CNS-equivalent roles (e.g., clinical nurse consultant, nurse consultant, advanced practice nurse roles without NP designation). Advanced practice nurses, such as Nurse Practitioners (NPs) and Clinical Nurse Specialists (CNSs), are graduate-prepared nurses with advanced clinical training and expanded scope of practice [4]. However, the NP and CNS roles differ in their primary functions, scope of autonomy, and focus within healthcare delivery. The NP role is primarily focused on direct patient care and typically includes autonomous clinical responsibilities such as advanced assessment, diagnosis, prescribing, and treatment management [4]. In contrast, CNS roles are more focused on indirect aspects of care, including consultation, education, leadership, quality improvement, and advancing clinical practice within healthcare systems [4]. However, given that distinctions between NP and CNS roles are not always clear, studies in which both NP and CNS roles are described will be retained during initial title and abstract screening and then assessed in full text to minimize the exclusion of relevant evidence. These studies will be excluded by reviewers if findings specific to NP roles cannot be distinguished from CNS-related data.

Additionally, the focus of this scoping review is strictly related to NP practice in general pediatric wards. Thus, sources conducted in specialized pediatric settings (i.e., pediatric intensive care units (PICUs), neonatal intensive care units (NICUs), oncology, and surgical units) will be excluded, as these environments involve distinct patient populations and care delivery models that differ from general pediatric inpatient wards. These settings typically manage patients with higher acuity, condition-specific needs, or procedural care requirements, which may not reflect the broader scope of general pediatric inpatient practice. Similarly, studies focused on ambulatory and/or outpatient care will be excluded, as these settings do not involve hospitalized patients and operate under different models of care delivery. There will be no restrictions on study design or publication type, or country of publication. Both peer-reviewed and grey literature (including conference proceedings, editorials, book chapters, and organizational reports) will be eligible for inclusion.

### 2.2. Search Strategy

The search strategy will aim to identify both published and unpublished literature on nurse practitioner practice in general pediatric ward settings. An initial limited search of CINAHL Ultimate via EBSCO Information Services (EBSCOhost) and MEDLINE (via EBSCOhost) was conducted to identify relevant articles and refine search terms (see Table 2). This search was accessed through the software website https://about.ebsco.com/products/research-databases, on 19 March 2026. Text words from titles and abstracts, as well as index terms used to describe the articles, informed the development of the full search strategy (see Appendix A). The search strategy has been peer-reviewed by a librarian using the PRESS framework [13].

The full search strategy will be applied across multiple databases, including Scopus, CINAHL, MEDLINE, PsycINFO, the Cochrane Database of Systematic Reviews, ProQuest and JBI Evidence Synthesis. Grey literature will be searched using the web-based search engine Google, limited to the first ten pages of results as suggested by the “Comprehensive Literature Searching in the Health Sciences Guide” developed by the University of Ottawa [14]. Eligible grey literature will include reports, policy documents, conference proceedings, and theses/dissertations. Blogs, news articles, opinion pieces, and commercial content will be excluded. Every grey literature search will be logged, documenting the date searched, numbers of results screened, and the number of potentially eligible records [14]. The potentially relevant records will be imported into Covidence for screening. Additionally, reference lists of included studies will be screened for additional sources. The search will be limited to studies published in English and French as these are the languages in which the research team members are fluent, and this study is not funded to support professional language translation. The search will also be limited from 2016 to the present to capture the most up to date evidence and reflect the current contemporary state of practice.

### 2.3. Study Selection

Following the search, all identified citations will be imported into Covidence, an online tool for scoping review management [15]. Duplicate records will be detected and removed. Two reviewers will independently screen titles, abstracts, and full texts against the inclusion criteria, with a pilot test conducted before screening to ensure consistency. Reasons for exclusion at the full-text stage will be documented. Any disagreements will be resolved by consensus or, if needed, a third arbitrator. In the context of this scoping review, Cohen’s Kappa statistics will not be run. The search and selection process will be described in detail and presented using a PRISMA-ScR flow diagram [16].

### 2.4. Data Extraction

Data will be extracted independently by two reviewers using a JBI-informed form (Table 3). The extraction form was developed using both the PCC framework and the implementation domain of the PEPPA framework to ensure that data collected are aligned with the review objectives and implementation-focused research questions [10,11].

The PEPPA framework is a well-established advanced practice nursing framework that supports the examination of APN role development, implementation, integration, and evaluation within healthcare systems [11]. By framing this review with the PEPPA framework, it allows the inclusion of an implementation science lens which can be useful in examining and determining factors reported in the literature that may influence NP role operationalization within general pediatric wards.

The extraction form will be formatted into Microsoft Excel, which will be the platform used to organize, summarize, and analyze data [17]. The form will capture key information relevant to the review questions, including (1) characteristics of included sources, (2) models of NP care, (3) scope of NP practice, (4) implementation determinants, and (5) reported outcomes. Not all data elements on the form are expected to be reported in every source.

A data dictionary has been created for reviewers to better understand data items in the extraction form if needed (Appendix B). The draft extraction form will be pilot-tested on a sample of five studies to ensure clarity, consistency, and comprehensiveness. The form may be refined during data extraction as needed, and any modifications will be reported in the scoping review. Discrepancies between reviewers will be resolved through discussion with a third reviewer. When necessary, authors of primary sources will be contacted to request missing or additional data.

The methodological quality of eligible studies will be independently assessed by two reviewers according to study design, using the critical appraisal tools developed by the Joanna Briggs Institute [18].

### 2.5. Data Analysis and Presentation

In accordance with JBI, a descriptive analysis of study characteristics (i.e., year, country, study design) will be performed using frequency counts and percentages [19]. These quantitative and descriptive results will be visually presented in summary tables. A basic descriptive analysis of qualitative data using deductive simple coding will also be completed to categorize data based on the PEPPA framework implementation constructs, including role clarity, stakeholder involvement, organizational support, collaboration, education, policy, funding, and evaluation processes [10]. Barriers and facilitators identified across studies will also be grouped into broader categories to identify patterns influencing NP role implementation within this setting. A narrative summary will be used to address the review questions and describe patterns and gaps across the literature. This may be accompanied with a presentation of data in tables and/or figures. The implications of the scoping review findings will be discussed narratively in Section 3 while also outlining the limitations [19].

## 3. Discussion

This protocol describes in detail the methodological approach for a scoping review aiming to map literature on the implementation and practice of Nurse Practitioner (NP) roles in general pediatric ward settings, guided by the implementation domain of the PEPPA framework. The review is anticipated to identify themes related to role development, stakeholder involvement, implementation processes, and evaluation strategies of NP roles in the context of general pediatric inpatient settings. This review will contribute to clinical practice, policy, and research by providing an implementation-focused synthesis of NP roles in general pediatric inpatient settings. Findings may inform future workforce planning, role development, and sustainable models of care for general pediatric inpatient teams. In addition, this review is intended to serve as foundational work for future initiatives aimed at developing an evidence-informed implementation model for NP roles on general pediatric inpatient wards. Potential limitations include restriction to English and French language sources as well as the exclusion of subspecialty and outpatient settings, which may limit the transferability to other clinical contexts outside of general pediatric ward settings.

The title and abstract screening is anticipated to require approximately 2–3 months for completion, while full-text screening is expected to take an additional 2 months. Depending on the number of eligible studies identified, data extraction is projected to require 2–3 months, followed by approximately 3 months for data analysis. Manuscript preparation is expected to require a further 4–6 months. This timeline has been developed in consideration of the authors’ limited protected research time available alongside their clinical responsibilities. Overall, completion of the scoping review is anticipated within approximately 12–13 months, with an expected completion date of June 2027.

## 4. Conclusions

By mapping how NP roles are implemented and sustained in general pediatric wards, this review is expected to advance nursing knowledge and strengthen evidence for advanced practice nursing by generating relevant evidence for advanced clinical practice, workforce development, leadership, and health policy.

## Figures and Tables

**Table 1:** Exploratory preliminary search, 2025.

Database	Search Strategy Used	Filters Applied	Results
CINAHL	(nurse practitioners or advanced practice registered nurse or advanced registered nurse practitioner) AND (pediatric or child or children or infant or adolescent) AND (hospital or acute setting or inpatient or ward)	Language: English, French; Period: 2015–2025; Peer-Reviewed	355
PsycINFO	(nurse practitioners or advanced practice registered nurse or advanced registered nurse practitioner) AND (pediatric or child or children or infant or adolescent) AND (hospital or acute setting or inpatient or ward)	Language: English, French; Period: 2015–2025; Peer-Reviewed	321
MEDLINE	(nurse practitioners or advanced practice registered nurse or advanced registered nurse practitioner) AND (pediatric or child or children or infant or adolescent) AND (hospital or acute setting or inpatient or ward)	Language: English, French; Period: 2015–2025; Peer-Reviewed	1152
Cochrane	(nurse practitioners or advanced practice registered nurse or advanced registered nurse practitioner) AND (pediatric or child or children or infant or adolescent) AND (hospital or acute setting or inpatient or ward)	Language: English, French; Period: 2015–2025; Peer-Reviewed	71
JBI	Pediatric Nurse Practitioner AND Hospital	Period: Last 10 years	16
OSF	Pediatric Nurse Practitioner AND Hospital	Period: Last 10 years	1

**Table 2:** Search strategy for EBSCOhost (CINAHL and MEDLINE).

Search	Search Terms
1	(MH “Pediatrics+”) OR (MH “Infant+”) OR (MH “Child+”) OR (MH “Adolescence+”) OR (MH “Young Adult+”) OR pediatric* OR paediatric* OR child* OR adolescen* OR “young adult*” OR infant* OR neonat*
2	(MH “Nurse Practitioners+”) OR (MH “ Pediatric Nursing+”) OR nurse practitioner* OR “pediatric nurse practitioner*” OR “pediatric acute care nurse practitioner*” OR “acute care nurse practitioner*” AND (role* OR “scope of practice” OR “model* of care” OR competenc* OR responsibilit*)
3	(MH “Hospitals, Pediatric+”) OR (MH “Inpatients+”) OR hospital* OR “children’s hospital*” OR “pediatric hospital*” OR “rehabilitation hospital” OR “rehabilitation unit” OR “short stay” OR “extended care” OR “observation medicine” OR “multidisciplinary team*”
4	#1 AND #2 AND #3
Limits of search	peer-reviewed articles, 1960 to present, English and French language

* = truncation to broaden the search and find variations of words.

**Table 3:** Data extraction tool.

Evidence Source Details and Characteristics	
Citation Details:	Title:
	Author(s):
Country:	□ Canada □ USA □ International (indicate country):
Language:	□ English □ French
Context/Setting:	□ Pediatric Acute Care Setting: □ Hospital (inpatient) □ Pediatric acute care unit □ Other:
Study Aim(s) (free text):	
Source type and study design (free text):	
Methods used:	□ Interviews □ Surveys □ Focus Groups □ Observation □ Chart reviews □ Other:
PEPPA Framework: Implementation Domain	
Population:	□ Hospitalized children (<18 years) □ Specific condition (e.g., cystic fibrosis, children with chronic illness, etc.): □ Data on equity, diversity, inclusion and Indigeneity (specify):
Model of Care:	□ Pediatrician/Physician only □ NP/APN only model □ Shared Care model (e.g., MD/NP): specify □ Details of NP role:
Stakeholders (check all that apply):	□ Management involvement □ Physician/pediatrician □ Multi-disciplinary team engagement (e.g., allied health) □ Community agencies □ Other:
Factors indicating need for model of care and improvement in goals (check all that apply)	□ Enhance care □ Decrease LOS □ Continuity of Care □ Specialized care needs □ Shortage of physicians □ Need for Multi-D model □ Other:
NP role identified in the article (free text input)	
Outcomes measured (patient, provider, and system-level)	□ Length of Stay □ Patient/parent satisfaction □ Medical staff satisfaction □ Time to complete consultation/care needs □ Cost Savings □ No outcomes were measured □ Other:
Implementation strategies (patient, provider, health system):	Facilitators: □ Stakeholder awareness and support of role □ NP education □ Resources to support implementation of role □ Policies and procedures developed □ Funding obtained □ Other: □ Barriers: □ Lack of Resources □ Lack of Support □ Other:
NP scope of practice	□ Autonomous practice and in coordination with MRP □ Domains of practice (as per ICN, Canada, AANP): □ Clinical □ Education □ Leadership □ Research □ Quality Improvement
Timeline/phases of implementation: (free text)	
Resources used for NP role implementation:	□ PEPPA framework □ Inspiration from other institutions □ Other:
Projected long-term monitoring of NP role and model of care	□ Sustained funding model □ Sustained Stakeholder support □ Acknowledged value of role/model of care □ Acceptability from other team members (e.g., pediatricians, allied health, multi-d team). □ Not reported □ Other:
Implications for Practice/Policy/Research:	□ Recommendation of role being beneficial in other settings □ Committing to further research □ Identifying further research is beneficial □ Other:
Notes/Additional Comments:	

## Data Availability

Data sharing is not applicable. No new data were created or analyzed in this study.

## Public Involvement Statement

No public involvement in any aspect of this research.

## Guidelines and Standards Statement

This manuscript was drafted against the Preferred Reporting Items for Systematic reviews and Meta-Analyses extension for Scoping Reviews (PRISMA-ScR) Checklist for scoping review research.

## Abbreviations

The following abbreviations are used in this manuscript:

NP	Nurse Practitioner
PEPPA	Participatory, Evidence-based, Patient-focused Process for Advanced Practice Nursing

## Appendix A

**Table A1:** Search Strategy Developed for Databases, 2026.

Database	Search Strategy
CINAHL MEDLINE PsycInfo Cochrane Via EBSCOhost	((MH “Pediatrics+”) OR (MH “Infant+”) OR (MH “Child+”) OR (MH “Adolescence+”) OR (MH “Young Adult+”) OR pediatric* OR paediatric* OR child* OR adolescen* OR “young adult*” OR infant* OR neonat*) AND ((MH “Nurse Practitioners+”) OR (MH “Pediatric Nursing+”) OR nurse practitioner* OR “pediatric nurse practitioner*” OR “pediatric acute care nurse practitioner*” OR “acute care nurse practitioner*” AND (role* OR “scope of practice” OR “model* of care” OR competenc* OR responsibilit*)) AND ((MH “Hospitals, Pediatric+”) OR (MH “Inpatients+”) OR hospital* OR “children’s hospital*” OR “pediatric hospital*” OR “rehabilitation hospital” OR “rehabilitation unit” OR “short stay” OR “extended care” OR “observation medicine” OR “multidisciplinary team*”)
Scopus Via Elsevier	TITLE-ABS-KEY (pediatric* OR paediatric* OR child* OR adolescen* OR “young adult*” OR infant* OR neonat*) AND (TITLE-ABS-KEY ((“nurse practitioner*” OR “pediatric nurse practitioner*” OR “paediatric nurse practitioner*” OR “pediatric acute care nurse practitioner*” OR “acute care nurse practitioner*”) AND (role* OR “scope of practice” OR “model* of care” OR competenc* OR responsibilit*))) AND (TITLE-ABS-KEY (hospital* OR “children’s hospital*” OR “pediatric hospital*” OR “paediatric hospital*” OR inpatient* OR “rehabilitation hospital*” OR “rehabilitation unit*” OR “short stay” OR “extended care” OR “observation medicine” OR “multidisciplinary team*”))
JBI Evidence Synthesis	Keywords: nurse practitioner, pediatrics, hospital
Google	((“nurse practitioner” OR “pediatric nurse practitioner” OR “paediatric nurse practitioner”) AND (“general pediatric” OR “general paediatric” OR inpatient OR hospital OR ward) AND (role OR “scope of practice” OR implementation OR “model of care”))
ProQuest	((pediatric* OR paediatric* OR child* OR adolescen* OR “young adult*” OR infant* OR neonat*) AND (“nurse practitioner*” OR “pediatric nurse practitioner*” OR “paediatric nurse practitioner*” OR “acute care nurse practitioner*”) AND (role* OR “scope of practice” OR “model* of care” OR competenc* OR responsibilit* OR implementation) AND (hospital* OR inpatient* OR ward* OR unit* OR “general pediatric ward*” OR “children’s hospital*”))
Limits of search: peer-reviewed articles published from 2016 to present, English and French language only.	

* = truncation to broaden the search and find variations of words.

## Appendix B

**Table A2:** Data Dictionary.

Data Items	Definitions
Citation Details: (Title and citation, authors)	The citation of the source following APA format. Author(s). (Year of publishing). Article Title. Journal Name, Volume (Issue), Page range. DOI or URL
Country:	The country in which the source was published.
Language	The language in which the source was published.
Context/Setting:	The healthcare setting that is discussed in the source (i.e., pediatric unit, hospital, ICU, NICU)
Study Aim(s):	The objective/aim of the study, described in free text.
Source type & Study design	The source type is divided between peer-reviewed or non-peer-reviewed sources. The process for peer-reviewed sources includes formal review of the source by experts in a specific field to assess the validity, quality, and originality of the source. Non-peer reviewed sources are not formally reviewed in the same process. The study design refers to the methodology/process chosen by the authors to answer the research question (i.e., quantitative descriptive design, interpretive description, phenomenology, meta-analysis, systematic review). The study design is often described in the methods section of the abstract/article. In non-peer reviewed or grey literature, this includes informal sources such book chapters, editorials, opinion pieces, or governmental/organizational reports.
Methods:	Methods are concrete tools that are used to collect data (i.e., individual interviews, focus groups, observation, chart reviews, surveys).
PEPPA Framework	
Define patient population	Define the target patient population, including any patient groups. There is no set definition for a patient population; however, examples include high-risk, high-cost, high-volume patient groups.
Model of Care	Describe the current model of care and the health care professionals that lead patient care.
Stakeholders	Stakeholders are key members of the team who have participated in the implementation process. Stakeholders can be both internal and external from the organization and model of care.
Factors indicating need for model of care and goals to improve	This related to the factors that contributed to the need for a role change and the need for the implementation of an NP. Some studies use needs assessments to identify these factors as well as existing use of health care services, gaps in health care services, and unmet patient needs.
Model of Care	Describe the new model of care and the NP role identified in the article. Components of NP practice include clinical practice, leadership, education, research, and professional development.
Outcomes measured in study (patient, provider, and system-level)	Outcomes relate to the impact and results of the proposed role. Outcomes can be described from the perspective of the health care system, organization, patients/families, other health providers, and the APN themselves. Examples include patient/family satisfaction, decreased wait times, and improved continuity of care.
Outcomes and implementation strategies (patient, provider, health system):	Identify the facilitators and barriers to successful implementation of the proposed NP role.
NP scope of practice	Describe the scope of practice of the NPs identified in the study. NP scope of practice is generally regulated by province or by country. If unclear, please refer to the official documents of the country of publication outlining their NP scope of practice. In general, NP scope of practice includes the following domains (as per ICN, Canadian and AANP Scopes of practice): Clinical—advanced health assessment, diagnosis, order and interpret diagnostic and lab tests, consult and refer to other health professionals; management that includes developing and sharing a management plan, prescribe pharmacological agents and nonpharmacologic therapies, provide patient education and counselling; evaluation of effectiveness of management plan and implement required modifications of treatment; professional responsibility and accountability for their practice. Education—developing and providing education to enhance knowledge, skills and build capacity; improve outcomes through evaluating learning and delivery methods. Leadership—contributing to development of high-quality healthcare system; supporting and creating a culture of improvement, safety and excellence; design, implement and evaluate health promotion and disease prevention programmes. Advocacy—promoting equitable and diverse care and service delivery; advocating for access to resources and system changes that demonstrates cultural safety; supporting development of policies and legislation for improving health; providing culturally safe care for indigenous peoples. Research and Qualitive Improvement—contributing to research initiatives for promoting evidence informed practice; promote knowledge translation of research findings for improving healthcare and systems outcomes.
Timeline/phases of implementation:	Describe the phases of the implementation process and associated duration.
Resources used for NP role implementation:	Identify the resources that were used for NP role implementation. Examples include organization and professional frameworks, literature, and inspiration.
Projected long-term monitoring of NP role and model of care	Identify the plan for long-term monitoring of the proposed NP role and associated model of care.
Implications for Practice/ Policy/Research:	Identify future direction because of the proposed NP role and model of care. Examples include recommendations for further research, or implementation of the NP roles in similar areas.
